# Safety of magnetic resonance imaging in patients with cardiac implantable electronic devices and abandoned or epicardial leads: a systematic review and meta-analysis

**DOI:** 10.1093/europace/euae165

**Published:** 2024-06-26

**Authors:** Claudia Meier, Carsten Israel, Michel Eisenblätter, Annika Hoyer, Ferdinand Valentin Stoye, Ali Yilmaz, Stephan Gielen

**Affiliations:** Campus Klinikum Lippe, Universitätsklinikum Ostwestfalen-Lippe, Universitätsklinik für Kardiologie, Angiologie und Internistische Intensivmedizin, Röntgenstraße 18, 32756 Detmold, Germany; Medizinische Fakultät, Universität Bielefeld, Postfach 10 01 31, 33501 Bielefeld, Germany; Klinik für Innere Medizin, Kardiologie, Nephrologie und Diabetologie, Evangelisches Klinikum Bethel, Bielefeld, Germany; Medizinische Fakultät, Universität Bielefeld, Postfach 10 01 31, 33501 Bielefeld, Germany; Campus Klinikum Lippe, Universitätsklinikum Ostwestfalen-Lippe, Universitätsinstitut für Diagnostische und Interventionelle Radiologie, Detmold, Germany; Medizinische Fakultät, Universität Bielefeld, Postfach 10 01 31, 33501 Bielefeld, Germany; Institut für Biostatistik und Medizinische Biometrie, Universität Bielefeld, Bielefeld, Germany; Medizinische Fakultät, Universität Bielefeld, Postfach 10 01 31, 33501 Bielefeld, Germany; Institut für Biostatistik und Medizinische Biometrie, Universität Bielefeld, Bielefeld, Germany; Herz-MRT-Zentrum, Universitätsklinikum Münster, Münster, Germany; Campus Klinikum Lippe, Universitätsklinikum Ostwestfalen-Lippe, Universitätsklinik für Kardiologie, Angiologie und Internistische Intensivmedizin, Röntgenstraße 18, 32756 Detmold, Germany; Medizinische Fakultät, Universität Bielefeld, Postfach 10 01 31, 33501 Bielefeld, Germany

**Keywords:** Magnetic resonance imaging (MRI), Cardiac implantable electronic devices (CIEDs), Abandoned leads, Epicardial electrodes, Safety, Tip heating

## Abstract

**Aims:**

Persistent reluctance to perform magnetic resonance imaging (MRI) in patients with abandoned and/or epicardial leads of cardiac implantable electronic devices is related to *in vitro* studies reporting tip heating. While there is a plethora of data on the safety of MRI in conditional and non-conditional implantable devices, there is a clear lack of safety data in patients with abandoned and/or epicardial leads.

**Methods and results:**

Relevant literature was identified in Medline and CINAHL using the key terms ‘magnetic resonance imaging’ AND ‘abandoned leads’ OR ‘epicardial leads’. Secondary literature and cross-references were supplemented. For reporting guidance, the Preferred Reporting Items for Systematic reviews and Meta-Analyses 2020 was used. International Prospective Register of Systematic Reviews (PROSPERO) registration number 465530. Twenty-one publications with a total of 656 patients with 854 abandoned and/or epicardial leads and 929 MRI scans of different anatomical regions were included. No scan-related major adverse cardiac event was documented, although the possibility of under-reporting of critical events in the literature should be considered. Furthermore, no severe device dysfunction or severe arrhythmia was reported. Mainly transient lead parameter changes were observed in 2.8% in the subgroup of patients with functional epicardial leads. As a possible correlate of myocardial affection, subjective sensations occurred mainly in the subgroup with abandoned epicardial leads (4.0%), but no change in myocardial biomarkers was observed.

**Conclusion:**

Existing publications did not report any relevant adverse events for MRI in patients with abandoned and/or epicardial leads if performed according to strict safety guidelines. However, a more rigorous risk–benefit calculation should be made for patients with epicardial leads.

## Graphical Abstract

**Graphical Abstract:**
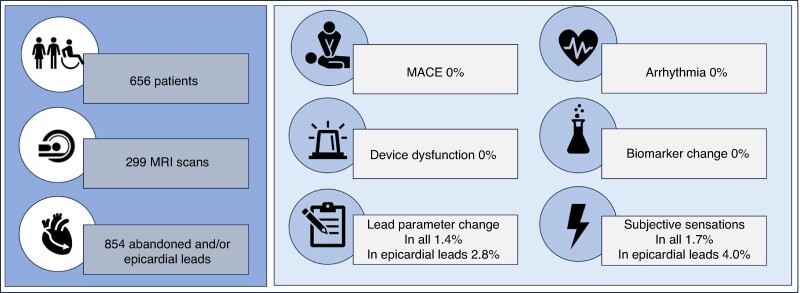

## Introduction

The indications for magnetic resonance imaging (MRI) of all anatomical regions are steadily increasing with advances in technology and medical science, and the importance of non-invasive, radiation-free examination methods is growing.^1^ At the same time, the number of patients with cardiac implantable electronic devices (CIEDs) is increasing as well. There are an estimated 4 million CIED patients worldwide and more than 500 000 new pacemakers and 100 000 new implantable cardioverter defibrillators (ICDs) are implanted in Europe per year.^2,3^ From a technical point of view, important safety aspects must be considered in patients with a CIED before, during, and after an MRI, such as mechanical, thermal, and therapeutic interactions due to the contact with the magnetic field and the absorption of high frequency energy.^4^ Therefore, the presence of a CIED was historically considered as a contraindication to MRI examination. Today, after 10 years of experience with MR-conditional labelled devices, MR scanning with CIED is well studied^2–10^ and established at many imaging centres under pre-defined safety conditions. Furthermore, data on formally non-conditional CIEDs are comprehensive and still growing^5,6^; in consequence, many national and international guidelines allow MRI even in non-conditional CIEDs under strict precautions.^7–15^

As the number and survival of device patients increases, the amount of so-called abandoned leads increases as well. Abandoned leads can be defined as electrodes that are not attached to a device generator and lead fragments remaining after unsuccessful lead extraction. They occur either in the presence of a functioning device with additional intact leads or after device removal in the absence of any electronic rhythmologic device.

In addition to abandoned intracardial leads, epicardial leads—frequently surgically implanted in case of device infections in case of device-dependent patients—are not MRI certified. Even when used with a conditional device, the entire system is considered non-conditional. If epicardial leads are inactivated, they may be called abandoned epicardial electrodes.

For both conditions—abandoned leads and epicardial leads (active and inactive)—the guidelines provide no or only limited recommendations due to a lack of data, as such patients were primarily not included in prospective studies for fear of tip heating, which has been reported in several *in vitro* studies.^16–27^ It should be noted that this meta-analysis included very different types of leads. As these types of leads are often categorized together in the guidelines, we decided to look at both types simultaneously. There is a clear need for a meta-analysis and a structured review to evaluate the safety of MRI in patients with abandoned and/or epicardial leads.

## Methods

### Publication search and selection

We used the medical scientific electronic databases Medline via PubMed and CINAHL to identify relevant peer-reviewed literature applying the key terms ‘magnetic resonance imaging’ AND ‘abandoned leads’ OR ‘epicardial leads’. Secondary literature and cross-references were searched manually and supplemented, and experts were contacted. The search was limited to studies in humans. A search was also conducted in German, French, Spanish, and Italian to find smaller case reports, but no other relevant publications were found in other languages than English. The last database search in English was performed on 16 October 2023.

Inclusion criteria were (i) enrolment of patients with abandoned and/or epicardial leads undergoing any kind of MRI and (ii) adverse events during or immediately after MRI was assessed. There was no restriction on the time of publication and no limitation for the study design. Case reports and case series were also included. All publications were screened manually and were excluded if titles and abstracts did not match the pre-defined topic. Of all potentially relevant publications, the full-text article was retrieved and evaluated independently by two reviewers for fulfilling all inclusion and exclusion criteria. Exclusion criteria were (i) comment to original data, (ii) *in vitro* data, (iii) review, (iv) lack of not specified central information (for example, no description of the electrode, the field strength or which outcome variables were observed), and (v) duplicated data (e.g. follow-up study). *In vitro* data were reviewed for discussion but not included in the meta-analysis. Post-surgical temporary epicardial leads that have been partially removed are not judged as abandoned or epicardial leads^9,28^ and thus are not included in the review. For studies that also included patients without abandoned/or epicardial leads, only information from patients of interest was extracted.

The primary outcome of interest in the selected studies was safety-relevant events, defined as (i) major adverse cardiac event (MACE; one of the following: death, inadequate ICD shock, total generator failure, and lead failure requiring operative intervention). Secondary outcome measures included (ii) major change in sensing or pacing parameters in functional epicardial and/or additional functional transvenous leads [increased pacing threshold >0,5 V or >50% compared with previous value, impedance change >50 Ohm or 30%, sensing reduction >30%, (these limit values were chosen on the basis of the heterogeneous limit value definitions of the cited studies)], (iii) device- or patient-related dysfunction (significant battery voltage reduction >20%, electrical reset, inappropriate pacing, and severe arrhythmia), and (iv) signs of potential myocardial affection (sensations, e.g. heating, pain, tingling, or pectoral discomfort) and/or laboratory measurement of myocardial tissue damage with biomarker (troponin and creatinine kinase). Bradycardia or pause in heart rhythm when programmed in a non-pacing mode during scan was not assessed as an adverse event because it was considered to be a programming decision and not a MRI-related event. All variables shown have been interpreted as a minimum due to missing or unspecified data. The presence of a cardiac resynchronization therapy was categorized as either pacemaker or ICD, depending on the generator.

The Preferred Reporting Items for Systematic reviews and Meta-Analyses (PRISMA) 2020 statement was used (PRISMA checklist available as Supplementary material online, *Data S1*), and this meta-analysis was registered on PROSPERO (International Prospective Register of Systematic Reviews, receipt 465530, review protocol can be accessed here). The Newcastle–Ottawa Scale (NOS) for cohort studies was used to assess study quality and potential bias based on the domains of subject selection, comparability, and assessment of outcome with a maximum possible score of 9 points by two independent reviewers. Greater than or equal to 7 points were considered as ‘good’, 3–6 points were considered as ‘fair’, and less than or equal to 2 points was considered as ‘poor’ quality. To improve the validity of this review, only studies with fair to good quality were included.

### Statistical analysis

Previously published results derived from the literature review were pooled and analysed by a meta-analysis to create a single, more precise estimate. Analysis and graphical presentation were performed in R Version 4.3.1^29^ using a forest plot and the R-package meta.^30^

For the meta-analysis, a beta-binomial model^31^ was applied to pool the outcome of interest that is defined as the proportion of reported symptoms (e.g. heating or pain) in relation to the number of scans. Other relevant outcomes, for example, MACE, could not be pooled in a meta-analysis as no events were observed. For statistical analysis, the beta-binomial model was particularly well suited as we had to include many studies reporting zero events in the meta-analysis. Regarding sensitivity analysis, the same analysis using a standard random effect meta-analysis model was performed.

## Results

**Figure 1:**
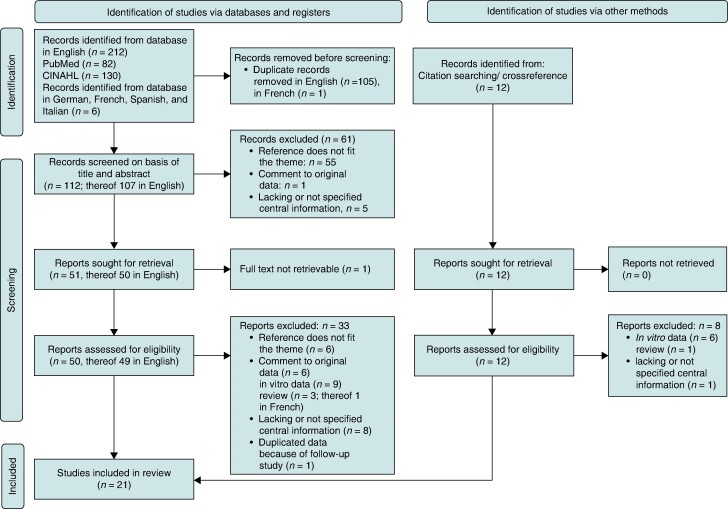
PRISMA flow chart.

A total of 218 publications were identified from the database, and 106 reports were removed immediately because of duplication (PRISMA diagram, *Figure 1*). The remaining 112 records were screened on the basis of title and abstract. A total of 61 did not meet all inclusion criteria. Among the 51 studies found by database search and 12 records, which were identified from citation searching, cross-reference, and hints from experts, 62 were considered for eligibility. After removing inappropriate studies because of exclusion criteria, 21 publications formed the final basis of the systematic review. Most studies also included patients without abandoned/or epicardial leads; hence, the subgroup information had to be extracted in those studies. The study characteristics are given in Supplementary material online, *Data S2*.^32–52^

We applied the meta-analysis to the endpoint ‘subjective sensation’ because this was the most frequently reported outcome. Therefore, those trials that did not report this outcome of interest also had to be excluded, resulting in 13 trials being used in the meta-analysis.

**Figure 2:**
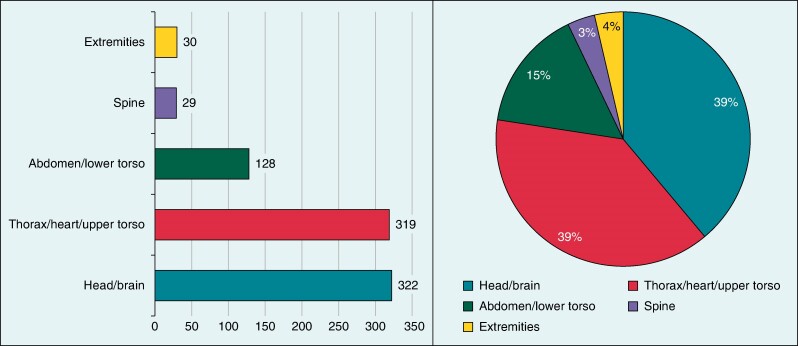
Anatomic region scanned.

In summary, 656 patients with 854 abandoned and/or epicardial leads underwent 929 MRI scans of different anatomical regions (*Figure 2*). With one exception,^41^ the publications all date from the last decade and contain data from 1990 to 2022.

Patients were predominantly male (69%) and had an average age of 45 years, and leads were in place for about 6.6 years at the time of scanning. Details in patients’ and device characteristics are illustrated in *Table 1*. In 39% of all MRIs, the thoracic region was scanned, so both the generator and the abandoned or epicardial electrodes were in the magnetic isocentre. All except two studies used a static field strength of 1.5 T with a specific absorption rate, specified as watts per kilogram (W/kg) limit ≤1.5 W/kg in four studies, ≤2 W/kg in five studies, and ≤4 W/kg in three studies. In 10 studies, the specific absorption rate level was not mentioned. The detailed settings of individual scan sequences were usually not specified due to their diversity and were therefore not recorded for this meta-analysis. Scanners by the manufacturers Philips, Siemens, and General Electrics were used.

**Table 1:** Patient and device characteristics

Population characteristics	Number^a^	
Number of patients, *n*	656	
Number of scans, *n*	929	
Number of abandoned/epicardial leads in total, *n*	854	
Number of abandoned/epicardial leads scanned, *n*	1011	
Device type specified in total, *n*	444 (thereof 65 without device generator)	
Device type specified in scans, *n*	753 (thereof 116 without device generator)	
Device and lead characteristics	Absolute number^a^	Number scanned^a^
Pacemaker, *n*	171	413
ICD, *n*	204	220
S-ICD, *n*	4	4
No generator, *n*	65	116
Abandoned transvenous pacing lead, atrial	100	108
Abandoned transvenous pacing lead, right ventricle	174	200
Abandoned transvenous HV/ICD lead, right ventricle	116	131
Abandoned transvenous pacing lead, coronary sinus	20	20
Functional epicardial lead	222	291
Abandoned epicardial lead	129	134
Functional subcutaneous array	2	3
Lead fragments	14	19

Cardiac resynchronization therapies have each been categorized as pacemaker or ICD, depending on the generator.

ICD, implantable cardioverter defibrillator; HV, high voltage/defibrillation; S-ICD, subcutaneous ICD.

^a^All numbers given are to be interpreted as minimum numbers, as the type of generators and/or leads was not always specified.

### Major adverse cardiovascular events

Scan-related MACEs were reported in 15 studies (*n* = 410 patients with *n* = 602 scans). These studies showed no MACE event during or immediately after the scan and during device interrogation. The remaining studies did not report any deaths or need for surgery, but the undefined statement ‘no adverse events’ was not considered to be a formally correct MACE report.

### Lead function changes

In 19 studies (*n* = 583 patients with *n* = 777 scans), changes in sensing or pacing parameters after scanning were reported. Unfortunately, lead function changes that might suggest tissue damage (e.g. pacing impedance) cannot be tested in abandoned but only in functional epicardial leads. In total, 11 (1.41%) lead function changes were documented in the entire study group. Eight changes occurred in patients with functional epicardial leads (three transient sensing decreases, two transient threshold increases, and three impedance increases). Two of the three impedance increases were irreversible but acceptable. One was interpreted as lead failure, but it occurred 6 months after scan, so a causal relationship to the previous MRI study was not really shown. In the evaluation of 285 scans with functional epicardial leads, the event rate of parameter changes was 2.81%. No need for device or lead replacement was reported in these patients.

### Device- or patient-related dysfunction

Device dysfunction, defined as inappropriate pacing (mentioned in *n* = 9 publications, *n* = 323 patients with *n* = 499 scans), battery voltage reduction (*n* = 15 publications, *n* = 423 patients with 596 scans), or electrical reset (*n* = 12 publications, *n* = 347 patients with 492 scans) was not observed in the study population. Except for transient bradycardia, when programmed in a non-pacing mode, no relevant arrhythmia events with a direct correlation to the abandoned or epicardial leads were reported (*n* = 9 publications, *n* = 326 patients with 462 scans).

### Signs of myocardial injury

Atypical sensations, reported by the patient and/or elevated levels of the biomarker troponin (cTNT) and myocardial creatinine kinase (CK-MB), are surrogate endpoints of myocardial injury. Two studies^36,52^ reported biomarker measurement (cTNT and/or CK-MB) with matched controls in a total of 40 abandoned and/or epicardial lead patients without a significant change before and after the scan, when compared with the respective control group.

Thirteen publications (*n* = 550 patients with *n* = 785 scans) documented subjective sensations of the patients, especially heating, pain, or tingling. In total, 13 events (1.65%) of subjective sensation of heating/pain occurred in the study population, mainly in patients with epicardial leads. In detail, five (4.03%) events happened in patients with abandoned epicardial leads, four (1.59%) events happened in functional epicardial leads, three (0.68%) events happened in abandoned endocardial leads, and one case in a patient with a subcutaneous array. Except for one case where impedance increase and warming occurred simultaneously, all adverse events occurred in different individuals. Details on outcome are listed in *Table 2* and Supplementary material online, *Data S3*.

**Table 2:** Outcome

	Cases present in publications, *n*	Patients included, *n*	Number of scans, *n*	Outcome occurred, *n*, (% related to number of scans^a^)
MACE	15	410	602	0 (0%)
Device parameter change (all) In functional epicardial leads	19 11	583 216	777 285	11 (1.42%) 8 (2.81%), thereof 3 permanent (1.05%)
Increased pacing threshold > 0,5 V or 50% (all) In functional epicardial leads				2 All in functional epicardial leads (0.70%)
Impedance change > 50 Ohm or 30% (all) In functional epicardial leads				3 All in functional epicardial leads (1.05%), all permanent
Sensing reduction > 30% (all) In functional epicardial leads				6 (0.77%) 3 in functional epicardial leads (1.05%) 3 in functional transvenous leads with an additional abandoned transvenous lead
Inappropriate pacing	9	323	499	0 (0%)
Significant battery voltage reduction > 20%	15	423	596	0 (0%)
Electrical reset	12	347	492	0 (0%)
Relevant arrhythmia	9	326	462	0 (0%)
Subjective sensations (all) In abandoned transvenous leads In abandoned epicardial leads In functional epicardial leads	13 11 8 5	550 399 119 184	785 441 124 251	13 (1.65%) 3 in abandoned transvenous leads (0.68%) 5 in abandoned epicardial leads (4.03%) 4 in functional epicardial leads (1.59%) 1 in subcutaneous array
Biomarker measurement (all) In epicardial leads	2 2	40 20	40 20	0 (0%) 0 (0%)

MACE, major adverse cardiac event.

^a^If subgroup is specified, reference value according to the subgroup.

**Figure 3:**
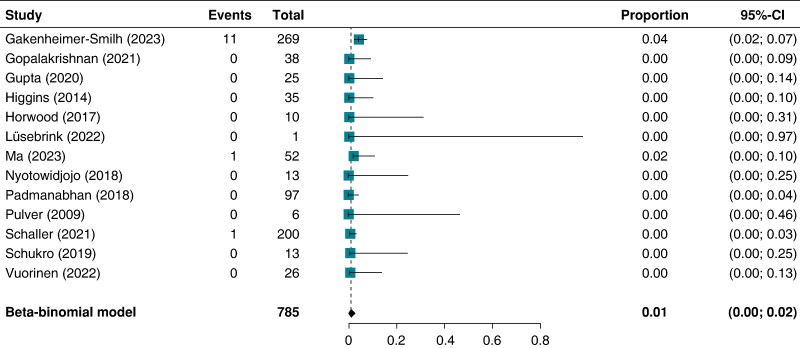
Forest plot beta-binominal model of the outcome ‘sensations’. CI, confidence interval.

Performing a meta-analysis using the beta-binomial model with the proportion of sensations in relation to the total number of scans as outcome of interest leads to an estimated proportion of 0.01 (95% confidence interval: 0.00, 0.02), indicating a rather small proportion of observed symptoms. This analysis was confirmed by applying the standard random effects model (see Supplementary material online, *Data S4*). *Figure 3* shows a forest plot, visualizing the individual study proportions of the 13 included studies along with their 95% confidence interval and the result of the meta-analysis.

### Quality of studies

The average NOS score of the included studies to assess internal and external validity aspects was six out of a possible nine points. No study was categorized as ‘poor’ and excluded regarding the quality. Five studies were rated ‘good’. Details on NOS are listed in Supplementary material online, *Data S5*.

## Discussion

The interactions of electromagnetic fields on CIEDs have been widely observed and studied for recent years. As a result, today we are living in an era of Food and Drug Administration-approved MR-conditional labelled CIEDs, and since 2017, there is a level of evidence Class IIa recommendation for performing MRI studies even in case of MR-non-conditional devices, provided safety guidelines are followed.^9^ This recommendation is supported by large studies and registries, such as the landmark publication of the MagnaSafe registry by Russo *et al*.^53^ as well as the large and comprehensive study by Nazarian *et al*.^54^ each covering about 1500 patients. Both studies were included in the meta-analysis from Munawar *et al*.^6^ with a total of over 5500 patients with non-conditional CIEDs and over 7000 MRI scans, in which not a single case of serious complications such as death or inappropriate shock delivery occurred.

However, the safety of MRI performed in patients with abandoned as well as epicardial leads remains largely unclear. This patient group was excluded in the comprehensive studies mentioned above.

Abandoned leads are a common problem, with an increasing trend as the population ages and CIEDs become more widespread. A large lead extraction study and data from ESC-EHRA European Lead Extraction ConTRolled Registry describe a prevalence of abandoned leads at 12–15%.^55,56^ With an estimated 4 million CIED carriers worldwide,^3^ this results in a prevalence of approximately 520 000 patients with abandoned leads. Assuming that up to 75% of CIED carriers will require an MRI at least once in their lifetime,^57^ this results in an estimated need for 390 000 MRIs with abandoned leads.

Reasons for lead abandonment are most often lead malfunction (56%), including lead fracture (15%), insulation defects (4%), dislocations (4%), and excessively high threshold values (32%).^58^ Therefore, endocardial implanted leads often remain in patients because extraction is difficult, dangerous due to adhesions and thus not recommended.^59^ Lead-related, operative re-intervention (extraction, repositioning, and new installation) occurs in 1.0–5.9%^13,60,61^ and lead extraction carries considerable risk of 0.4–2% procedural mortality.^62^ The removal of epicardial leads would always require thoracic surgery with associated risks. This is especially relevant for the paediatric population since abandoned leads will remain in the body for the rest of the lives of children with implanted epicardial pacing systems.

### Physical aspects and *in vitro* data

For a 1.5 T scanner, the radio frequency electric field has a frequency of 64 MHz, which corresponds to a wavelength of 0.52 m in water (representative for the human body),^20^ so resonance can occur because the common pacemaker- or ICD-lead lengths are of the same order (40–60 cm). The electrode then acts as an antenna, picking up the radio frequency field signal, amplifying it locally and transferring its energy to the tip of the electrode, potentially causing thermal damage to the heart muscle tissue.^25^ In case of abandoned leads, the termination condition at the connector is changing from a short circuit to an open circuit and do not dissipate energy through the generator. Therefore, abandoned leads are considered to significantly increase the risk of thermal injury and require even more careful consideration on the individual risk assessment.

The effects of MRI on CIED are complex and difficult to study systematically. Several theoretical, phantom, and animal studies have investigated the behaviour of the leads and generator. Selected studies and main findings are discussed in Supplementary material online, *Data S6*.^16–27^ Proof-of-concept studies have confirmed the potential role of computer models to predict the changes in pacing capture threshold and the degree of radio frequency-induced heating. Such computer-based simulations may aid to better assess safety and patient-specific outcomes in the future. Nonetheless, the individual circumstances and special conditions of a patient and his/her CIED and (abandoned) electrodes are so diverse that the respective physical interactions during MRI cannot be exactly predicted to such a degree that entirely excludes physically measurable and clinically relevant consequences.

### Implications from data analysis

Interestingly, the well-known theoretical concerns of myocardial injury by potential heating of the (abandoned or epicardial) electrode tip that are based on *in vitro* data are not supported by these clinical results. Two case–control studies^36,52^ included in this meta-analysis reported biomarker measurements (cTNT and/or CK-MB) without a significant change, which is the best approximation to reflect and assess injury, since no histopathological data are available for humans. The subjective feeling of heating as a correlate for tissue damage remains uncertain because heat sensation by the applied radiofrequency pulses and peripheral nerve stimulation by the time-varying gradients are common side effects of MRI. Heating-related issues in patients without CIED were reported in up to 1.48% of routine MRI scans,^63^ which is comparable with our present data (1.65%). It must be mentioned that the subjective sensation of heating showed a frequency trend in patients with abandoned epicardial leads (4.0%), but with such a low event rate, the significance should be questioned. The stronger subjective perception may be explained by stronger heating due to other physical properties, larger contact surface of epicardial electrodes compared with transvenous electrodes, and the lack of cooling blood flow or by different innervation of endocardium and epicardium.^64^

Another marker for cardiomyocyte death could be a deterioration of the measured lead-related parameters, especially signal sensing decrease and pacing threshold increase. Obviously, this can only be measured for functional electrodes. Significant parameter change was seen in up to 1.5% of endocardial leads in a large meta-analysis,^6^ which is similar to our findings in the total population. It remains unclear to what extent the changes are really associated with the MRI scan or at least partly subject to random variation of lead measurements, because it was shown that CIED patients undergoing cardiovascular MR showed comparable changes of CIED function during follow-up as CIED patients not undergoing any MR examination.^65^ However, it should be noted that subjective sensations and lead parameter changes were predominantly seen in the subgroup of epicardial functional leads in this study.

Second, there seems to be no adverse effect on the CIED generator, which might have occurred due to unpredictable, electromechanical interaction with the abandoned lead. Thirty-nine percent of the scans were located in the thorax, although some MR-conditional MR generators still have an exclusion zone for isocentre in the upper torso. No inappropriate pacing, significant battery voltage reduction, electrical reset, or other rare cause for periprocedural, premature device replacement was observed in our meta-analysis. This is in line with other studies, which found a low incidence of electrical reset (1.43%), inappropriate pacing (0.37%), decrease in battery voltage of >0.02 V (2.2%), or generator failure (0.14%) in patients with a non-conditional device.^6^

In summary, the present analysis of more than 900 MRI examinations did not reveal any serious adverse effects in patients with abandoned and/or epicardial leads, although the possibility of underreporting of critical events in the literature should be considered.

### Limitations

This meta-analysis was limited by the low number of available randomized clinical studies (only two such studies)—forcing us to also include observational studies and case reports. Generally, the quality of reporting MRI exposure was inconsistent with a lack of reporting on single or multiple MRI scans in the primary data. Reporting of the setting of abandoned leads (either with or without active devices) was inconsistent as well: Of the 21 included studies, 6 did not specify whether there were cases of abandoned leads without an active device while 7 trials only had cases with an active generator. In addition, in studies including cases without active device, no distinction was made in the outcome data and measurements between cases with and without generators.

There is a high likelihood of reporting bias as this is an off-label procedure, and adverse events may not be published. Significant heterogeneity was evident in the variables, e.g. the information, whether the number/type of electrodes was reported per patient or per scan, the various MRI scanner and sequence parameters, or different anatomic regions scanned.

From most studies, only data from subgroups were used for this meta-analysis, so that a total number of variables were available, but often no exact specification was provided. In addition, some of the studies have a small number of participants and we included case reports to collect the largest possible amount of available data, but this may underestimate the incidence of adverse events. The required information on the pre-defined variables and characteristics was not always available. Because not every potential lead and patient condition was tested in these studies, results cannot be generalized to all clinical situations and a careful individual risk–benefit decision is required. To date, there are only a few studies that have investigated the safety of CIED at a field strength of 3 T, and these are generally without abandoned or epicardial leads.^66^ Only one study of this meta-analysis also reported cases that were examined at 3 T, so that the statements made here are only valid for a field strength of 1.5 T and will have to be re-evaluated at higher field strengths in the future. Since randomized studies for research purposes are ethically difficult to justify in these patients, at least large, prospective case–control studies, with e.g. biomarker measurement, would be desirable. It would be useful to have large registries, like the EURObservational Registry Programme^67^ for the imaging data of patients with CIED. Last but not least, is well known that statistical significance is highly dependent on the number of events, so this criterion plays an important role in the evaluation of evidence. However, as there are very few adverse events reported in literature, this review remains the best reference for risk stratification.

## Conclusions

Physicians should always be aware that MRI safety protocol deviations carry a risk of serious adverse events, when performing off-label MRI scans in CIED patients. Appropriate alternative safe imaging modalities should be prioritized in patients with abandoned or epicardial leads (with or without active devices in place).

However, considering the limitations, based on this meta-analysis, which included 656 patients with 854 abandoned or epicardial leads and 929 MRI scans, no serious adverse event was published in the literature. Only few events of subjective heating or parameter change occurred as a correlate for possible but not proven myocardial damage particularly in patients with epicardial leads. Based on the available data, caution and a comprehensive risk–benefit assessment are recommended, especially in functional and abandoned epicardial leads. In patients with abandoned transvenous leads, a clinically indicated and carefully performed MRI at 1.5 T seems to be safe and feasible.

Well-designed large-scale registries on MRI safety in patients with abandoned and epicardial leads are still needed to develop clear guidelines in whom MRIs may be safely performed.

## Supplementary Material

euae165_Supplementary_Data

## Data Availability

The data underlying this article will be shared upon reasonable request to the corresponding author.
