# Supplementary material for: Safety of magnetic resonance imaging in patients with cardiac implantable electronic devices and abandoned or epicardial leads: a systematic review and meta-analysis
Source: Europace. 2024 Jun 26;26(6):euae165. doi: 10.1093/europace/euae165 (PMC11200101; doi:10.1093/europace/euae165)
Supplement: euae165_Supplementary_Data [file euae165_supplementary_data.zip › Supplement 2 Study characteristics.docx]

Supplement 2 Study characteristics

| First author | Date of publication | Data collection episode | Study design | Number of patients * | Median age* | Male* | Number of scans* | Number of abandoned or epicardial leads* | Tesla | Upper SAR ≤ | Scanner manufacturer |
| --- | --- | --- | --- | --- | --- | --- | --- | --- | --- | --- | --- |
| Schaller et al. ^32^ | 2021 | 2013 - 2020 | retrospective | 139 | 65 | 110 | 200 | 243 | 1,5 | 2 | na |
| Vuorinen et al. ^33^ | 2022 | 2011 - 2019 | retrospective | 17 | 37 | 10 | 26 | 24 | 1,5 | na | Siemens |
| Lüsebrink et al. ^34^ | 2022 | na | case report | 1 | 85 | 1 | 1 | 2 | 1,5 | na | Philips |
| Morris et al. ^35^ | 2018 | 2016 - 2017 | retrospective | 9 | 66 | 8 | 9 | 9 | 1,5 | 2 | General Electrics |
| Padmanabhan et al. ^36^ | 2018 | 2008 - 2017 | prospective | 80 | 66 | 53 | 97 | 90 | 1,5 | 1,5 | na |
| Higgins et al. ^37^ | 2014 | 1990 - 2013 | retrospective | 19 | 57 | 11 | 35 | 32 | 1,5 | 1,5 | na |
| Schukro et al. ^38^ | 2019 | 4 years | retrospective | 13 | ns | ns | 13 | 14 | 0,2 | 2 and 4 | Siemens |
| Hwang et al. ^39^ | 2016 | 1992 - 2015 | retrospective | 10 | ns | ns | 18 | 10 | 1,5 and 3 | na | na |
| Horwood et al. ^40^ | 2017 | na | retrospective | 10 | ns | ns | 10 | 12 | 1,5 | 2 | General Electrics |
| Pulver et al. ^41^ | 2009 | 2007 - 2008 | retrospective | 6 | 126 | ns | 6 | 9 | 1,5 | na | General Electrics |
| Gupta et al. ^42^ | 2020 | 2015 - 2019 | prospective | 15 | 68 | ns | 25 | 24 | 1,5 | na | General Electrics |
| Bireley et al. ^43^ | 2020 | 2010 - 2018 | retrospective | 17 | ns | ns | 44 | 17 | 1,5 | na | Philips and Siemens |
| Kulach et al. ^44^ | 2020 | 2017 - 2018 | case report | 1 | 68 | 1 | 2 | 2 | 1,5 | 1,5 | General Electrics |
| Pierpoline et al. ^49^ | 2020 | na | prospective | 15 | 63 | 10 | 17 | 21 | 1,5 | na | na |
| Ramirez-Suarez et al. ^45^ | 2022 | na | prospective | 5 | 15 | 5 | 5 | 9 | 1,5 | na | Siemens |
| Ma et al. ^52^ | 2023 | 2008 - 2021 | prospective | 29 | 43 | 12 | 52 | 66 | 1,5 | 1,5 | na |
| Gakenheimer-Smith et al. ^46^ | 2023 | 2007 - 2022 | retrospective | 186 | 15 | 139 | 269 | 186 | 1,5 | na | Siemens |
| Bhuva et al. ^47^ | 2022 | 2014 - 2019 | retrospective | 43 | ns | ns | 43 | 43 | 1,5 | 2 | General Electrics |
| Gopalakrishnan et al. ^48^ | 2021 | 2006 - 2019 | retrospective | 22 | ns | ns | 38 | 22 | 1,5 | < 4 | Philips and General Electrics |
| Bertelsen et al. ^50^ | 2017 | 2010 - 2013 | retrospective | 6 | ns | ns | 6 | 6 | 1,5 | 4 | Siemens |
| Nyotowidjojo et al. ^51^ | 2018 | 2013 - 2016 | retrospective | 13 | ns | ns | 13 | 13 | 1,5 | na | na |

*extracted subgroup, relevant for meta-analysis

Abbreviations: na = not available, ns = not specified, SAR = specific absorption rate
