# Supplementary material for: Safety of magnetic resonance imaging in patients with cardiac implantable electronic devices and abandoned or epicardial leads: a systematic review and meta-analysis
Source: Europace. 2024 Jun 26;26(6):euae165. doi: 10.1093/europace/euae165 (PMC11200101; doi:10.1093/europace/euae165)
Supplement: euae165_Supplementary_Data [file euae165_supplementary_data.zip › Supplement 3 Details in outcome.docx]

Supplement 3: Details of outcome


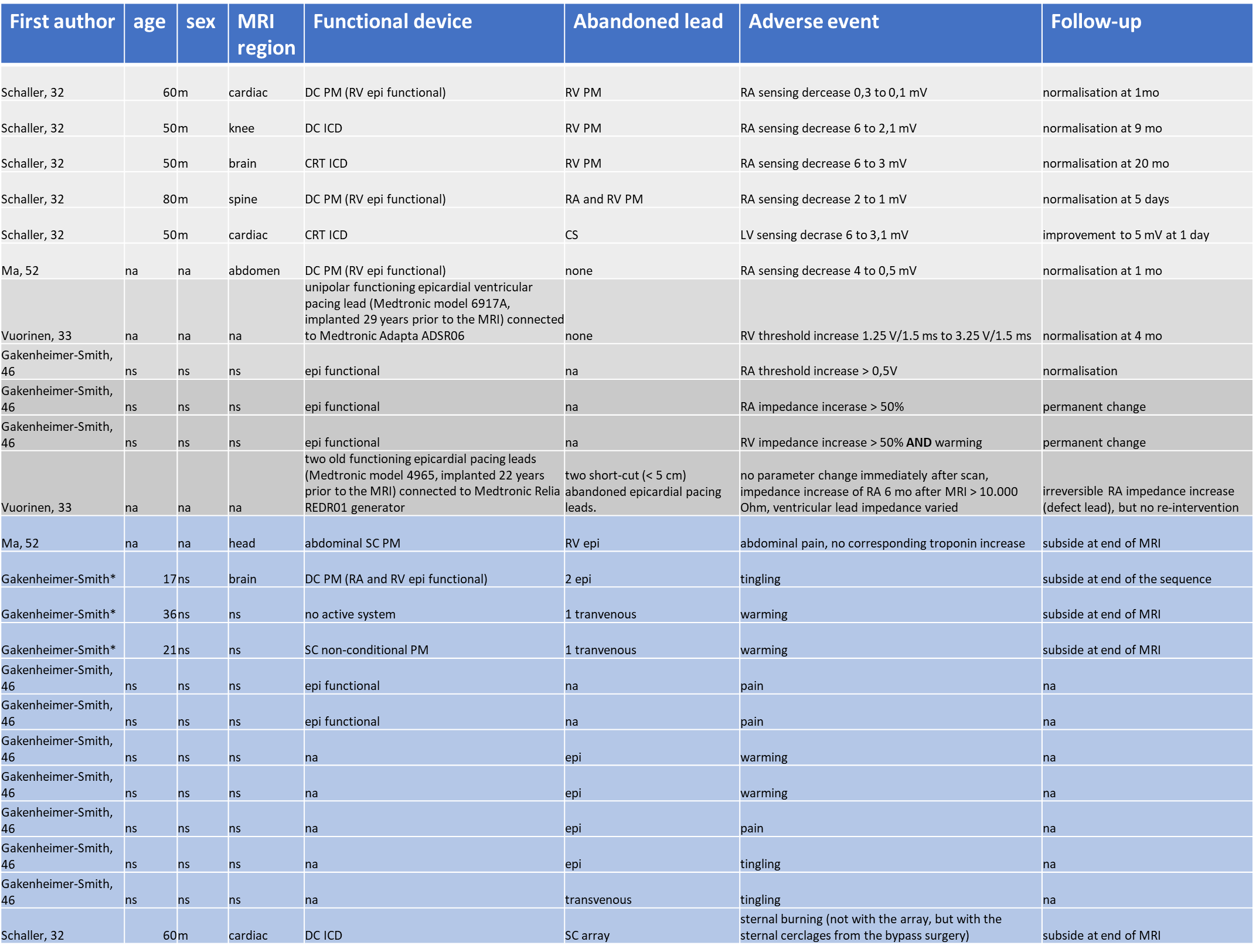


* Detailed information from previous study: Gakenheimer-Smith L, Etheridge SP, Niu MC, Ou Z, Presson AP, Whitaker P, Su J, Puchalski MD, Asaki SY, Pilcher T. MRI in pediatric and congenital heart disease patients with CIEDs and epicardial or abandoned leads. *Pacing Clin Electrophysiol* 2020;**43**:797–804.

Abbreviations: m = male, DC = dual chamber, PM = pacemaker, RV = right ventricle, epi = epicardial, RA = right atrium, mo = month, ICD = implantable cardioverter defibrillator, CRT = cardiac resynchronization therapy, CS = coronary sinus, LV = left ventricle, na = information not available, ns = information not specified, SC = subcutaneous
