# Supplementary material for: Safety of magnetic resonance imaging in patients with cardiac implantable electronic devices and abandoned or epicardial leads: a systematic review and meta-analysis
Source: Europace. 2024 Jun 26;26(6):euae165. doi: 10.1093/europace/euae165 (PMC11200101; doi:10.1093/europace/euae165)
Supplement: euae165_Supplementary_Data [file euae165_supplementary_data.zip › Supplement 6 non-human data.pdf]

## Supplement 6: In vitro/ non human data

The established method to evaluate the tip heating is described in the ISO (the International Organization for Standardization) statement ISO/TS 10974:2018(en)[1].

The following safety aspects have been described in animal or in vitro studies.

(1) A definitive thermal rise can be expected, when electrodes with and without CIED are examined on clinically used MRI-sequences, due to the absorbed HF-energy. Epicardial leads seem to get hotter, regardless whether they are connected to a generator or not [2,3]. Temperature rise between 2,5° - 20° degrees Celsius could be detected in functional, transvenous, attached pacing systems [2,4,5]. The degree of tissue damage depends on the amount and duration of the applied energy. Electrophysiological studies show, that immediate and irreversible cardiomyocyte cell death can be assumed from approximately 50 °C degrees Celsius after a 60-second hyperthermic exposure [6] (i.e. an increase in temperature of approx. 13°, starting from a normal body temperature) and other suggested a limit of 6°C for 26 minutes to induce any measurable effect in muscles [7]. Therefore, it is not possible to make a general statement about the potential for tissue damage in view of the many different MRI sequences. However, histopathological analysis in animal studies revealed no or very limited necrosis or fibrosis around the tip of the lead, which was not different from findings in control implants not having a MRI examination [5,8].

(2) The already reported extent of heating varies greatly from 0,2°[8] up to 90° degrees Celsius [9], as heating is obviously highly dependent on environmental factors and the conductive medium. Interestingly, it has been demonstrated, that a metal-capped abandoned lead shows lower heating than the complete CIED system, but a plastic-capped lead – as performed routinely – shows a higher rise of temperature [10]. One hypothesis is, that the major reason for not heating up in vivo is the cooling effect of blood flow through the heart tissue and around the lead tip–tissue interface [9]. Furthermore it could be shown, that the heating effects were much lower when the electrodes were inserted in saline [9], gel [4] or tissue [8]. In addition, presence of another intact, or even broken wire was seen to reduce the potential enhancement because of electromagnetic coupling [11].

(3) The position and length of the generator and the electrodes have a significant influence on the physical interactions as well [12,13]. This explains why both, increasing and decreasing temperature was observed with longer and shorter lead length [2–4] and one group found significant enhancement of lead-tip power in a broken wire [11], while another team only observed a low temperature rise in fragmented leads[14].

(4) Of course, the absorbed radiofrequency energy directly depends on static magnetic field strength (e.g. 1.5 T), spatial gradient, time-varying magnetic field (dB/dt), radiofrequency (RF) fields, and specific absorption rate (SAR) the total length of the examination and on the region of imaging, so RF-related heating of a metallic device is also reduced with increasing distance from the imaging landmark [15].

A short note is given to the terms “abandoned” and “retained” leads. Abandoned leads are not attached to the CIED generator anymore and left (often capped) in the body with the tip in contact with cardiac tissue. Retained leads had an attempt of extraction, are often removed from implantation place and shortened, so they are therefore no longer in contact with the cardiac tissue. On the one hand, there is no risk of myocardial injury at the tip, but on the other hand, the electromagnetic interactions are even less predictable.

References:


1. ISO/TS 10974:2018(en), Assessment of the safety of magnetic resonance imaging for patients with an active implantable medical device. https://www.iso.org/obp/ui/en/#iso:std:iso:ts:10974:ed-2:v1:en (2 October 2023)

2. Balmer C, Gass M, Dave H, Duru F, Luechinger R. Magnetic resonance imaging of patients with epicardial leads: in vitro evaluation of temperature changes at the lead tip. *J Interv Card Electrophysiol* 2019;**56**:321–6.

3. Jiang F, Henry KR, Bhusal B, Sanpitak P, Webster G, Popescu A, *et al.* Age Matters: A Comparative Study of RF Heating of Epicardial and Endocardial Electronic Devices in Pediatric and Adult Phantoms during Cardiothoracic MRI. *Diagnostics (Basel)* 2023;**13**:2847.

4. Langman DA, Goldberg IB, Finn JP, Ennis DB. Pacemaker lead tip heating in abandoned and pacemaker-attached leads at 1.5 Tesla MRI. *J Magn Reson Imaging* 2011;**33**:426–31.

5. Luechinger R, Zeijlemaker VA, Pedersen EM, Mortensen P, Falk E, Duru F, *et al.* In vivo heating of pacemaker leads during magnetic resonance imaging. *Eur Heart J* 2005;**26**:376–83; discussion 325-327.

6. Haines DE. Letter by Haines Regarding Article, “Direct Measurement of the Lethal Isotherm for Radiofrequency Ablation of Myocardial Tissue”. *Circulation: Arrhythmia and Electrophysiology* American Heart Association; 2011;**4**:e67–e67.

7. Ps Y, Ej M, C L, A M, Dw H, Bl V, *et al.* Thresholds for thermal damage to normal tissues: an update. *International journal of hyperthermia : the official journal of European Society for Hyperthermic Oncology, North American Hyperthermia Group* Int J Hyperthermia; 2011;**27**.

8. Roguin A, Zviman MM, Meininger GR, Rodrigues ER, Dickfeld TM, Bluemke DA, *et al.* Modern Pacemaker and Implantable Cardioverter/Defibrillator Systems Can Be Magnetic Resonance Imaging Safe. *Circulation* 2004;**110**:475–82.

9. Achenbach S, Moshage W, Diem B, Bieberle T, Schibgilla V, Bachmann K. Effects of magnetic resonance imaging on cardiac pacemakers and electrodes. *Am Heart J* 1997;**134**:467–73.

10. Wang. Magnetic resonance conditionality of abandoned leads from active implantable medical devices at 1.5 T.

11. Yao A, Goren T, Samaras T, Kuster N, Kainz W. Radiofrequency-induced heating of broken and abandoned implant leads during magnetic resonance examinations. *Magn Reson Med* 2021;**86**:2156–64.

12. Mattei E, Gentili G, Censi F, Triventi M, Calcagnini G. Impact of capped and uncapped abandoned leads on the heating of an MR-conditional pacemaker implant. *Magn Reson Med* 2015;**73**:390–400.

13. Bhusal B, Jiang F, Kim D, Hong K, Monge MC, Webster G, *et al.* The Position and Orientation of the Pulse Generator Affects MRI RF Heating of Epicardial Leads in Children. *Annu Int Conf IEEE Eng Med Biol Soc* 2022;**2022**:5000–3.

14. Nguyen BT, Bhusal B, Rahsepar AA, Fawcett K, Lin S, Marks DS, *et al.* Safety of MRI in patients with retained cardiac leads. *Magn Reson Med* 2022;**87**:2464–80.

15. Nordbeck P, Ritter O, Weiss I, Warmuth M, Gensler D, Burkard N, *et al.* Impact of imaging landmark on the risk of MRI-related heating near implanted medical devices like cardiac pacemaker leads. *Magn Reson Med* 2011;**65**:44–50.

Selected in vitro/ non-human in vivo data and main findings (ordered by date of publication)

| Reference | Main finding |
|---|---|
| Yao et al. 2020 | "In a case of a wire break the lead-tip power reaches 30-fold under isoelectric conditions (in vitro) and 16-fold in realistic clinical exposures (in vivo), compared to the intact lead. Because of electromagnetic coupling, the presence of a nearby intact, or even broken, wire was seen to reduce the potential enhancement significantly, to ~4 times for the nearby intact wire and ~7 times for the nearby broken wire, respectively." |
| Wang et al. 2021 | "RF-induced lead-tip heating of abandoned leads strongly depends on the proximal lead termination. Leads proximally capped with metal is lower than that from the complete CIED system. Leads proximally insulated with plastic caps could lead to temperature rise up to 3.5 times higher than that from the complete AIMD system." |
| Balmer et al. 2019 | "A temperature rise of + 2.5 °C was observed for the transvenous lead attached to an MRI-conditional pacemaker. The epicardial lead attached to the same pacemaker showed four times higher heating. The transvenous lead without pacemaker showed four times higher heating, and the epicardial lead without pacemaker showed 30 times higher heating. The epicardial lead coiled to 20 cm length without pacemaker showed 9 times higher heating. Experiments with various lengths of epicardial leads showed that the shorter the leads were, the smaller was the heating effect." |
| Mattei et al. 2014 | "Given a whole-body SAR = 1 W/kg, a maximum temperature rise of 17.6°C was observed. The presence of the abandoned lead modifies the RF-heating profile of the MR-conditional implant: either an increase or a decrease in the induced heating at its lead tip can occur, mainly depending on the relative position of the two leads. Variations ranging from −63% to +69% with respect to the MR-conditional system alone were observed." |
| Langman et al. 2011 | "The pacemaker-attached lead exhibited maximum heating (11.6) at a lead length of 20 cm and minimum heating (1.3) at 60 cm, exhibiting decreasing heating with increasing lead length. The abandoned-capped lead exhibited maximum heating (29.9) at a lead length of 60 cm and minimum (1.9) heating at 20 cm, exhibiting increasing heating with decreasing lead length. The abandoned gel-exposed lead exhibited maximum heating (12.0) at a lead length of 40 cm and minimum (2.2) heating at 20 cm, exhibiting a peak heating response at 40 cm with decreased heating for both shorter and longer lead lengths." |
| Jiang et al. 2023 | "(…) significantly higher RF heating of epicardial leads than endocardial leads in the pediatric phantom, but not in the adult phantom. Additionally, body size and lead length significantly affected RF heating, with RF heating up to 12 ◦C observed in models based on younger children with short epicardial leads." |
| Bhusal et al. 2022 | "Changing the orientation and position of the generator resulted in a five-fold variation in the RF heating at the lead's tip." |
| Nguyen et al. 2021 | "The maximum temperature rise when fragmented retained leads were positioned at the location of maximum electric field exposure was measured to be 2.4°C at 3 T and 2.1°C at 1.5 T." |
| Nordbeck et al. 2011 | "(…) the overall potential of implanted devices to develop severe RF-related heating is also reduced with increasing distance from the imaging landmark." |
| Achenbach et al. 1997 | "We recorded maximum temperatures of almost 90° C, and myocardial necrosis could be demonstrated in histologic studies, but there were large differences between the different electrodes: only seven electrodes showed an increase in temperature of more than 15 ° C during 90 seconds of imaging. The effects were attenuated when a pacemaker was connected to the electrodes and the electrodes were submersed in saline solution." |
| Roguin et al. 2004 | "Lead temperatures in vitro increased 1.5°C to 5.7°C for pacemaker leads and 0.2°C to 7.2°C for ICD leads tested. For in vivo comparisons, with leads placed in the right ventricle of dogs with acute implantation, the maximal heating was 0.2°C, even with the maximal energy protocol. In all 15 animals that were scanned after chronic implantation, histopathological analysis revealed no or very limited necrosis or fibrosis around the tip of the lead, which was not different from findings in control animals (n=3) not undergoing MR scans. " |
| Luechinger et al. 2005 | "Temperature increases of up to 20 degrees C were measured during MRI of the heart" (at 1,5T in pigs with endocardial PM leads, attached to generator). "Significant impedance and minor stimulation threshold changes could be seen. Pathology and histology could not clearly demonstrate heat-induced damage." |
