# Supplementary figures and images for: Safety of magnetic resonance imaging in patients with cardiac implantable electronic devices and abandoned or epicardial leads: a systematic review and meta-analysis
Source: Europace. 2024 Jun 26;26(6):euae165. doi: 10.1093/europace/euae165 (PMC11200101; doi:10.1093/europace/euae165)

Supplement 1: PRISMA check lists


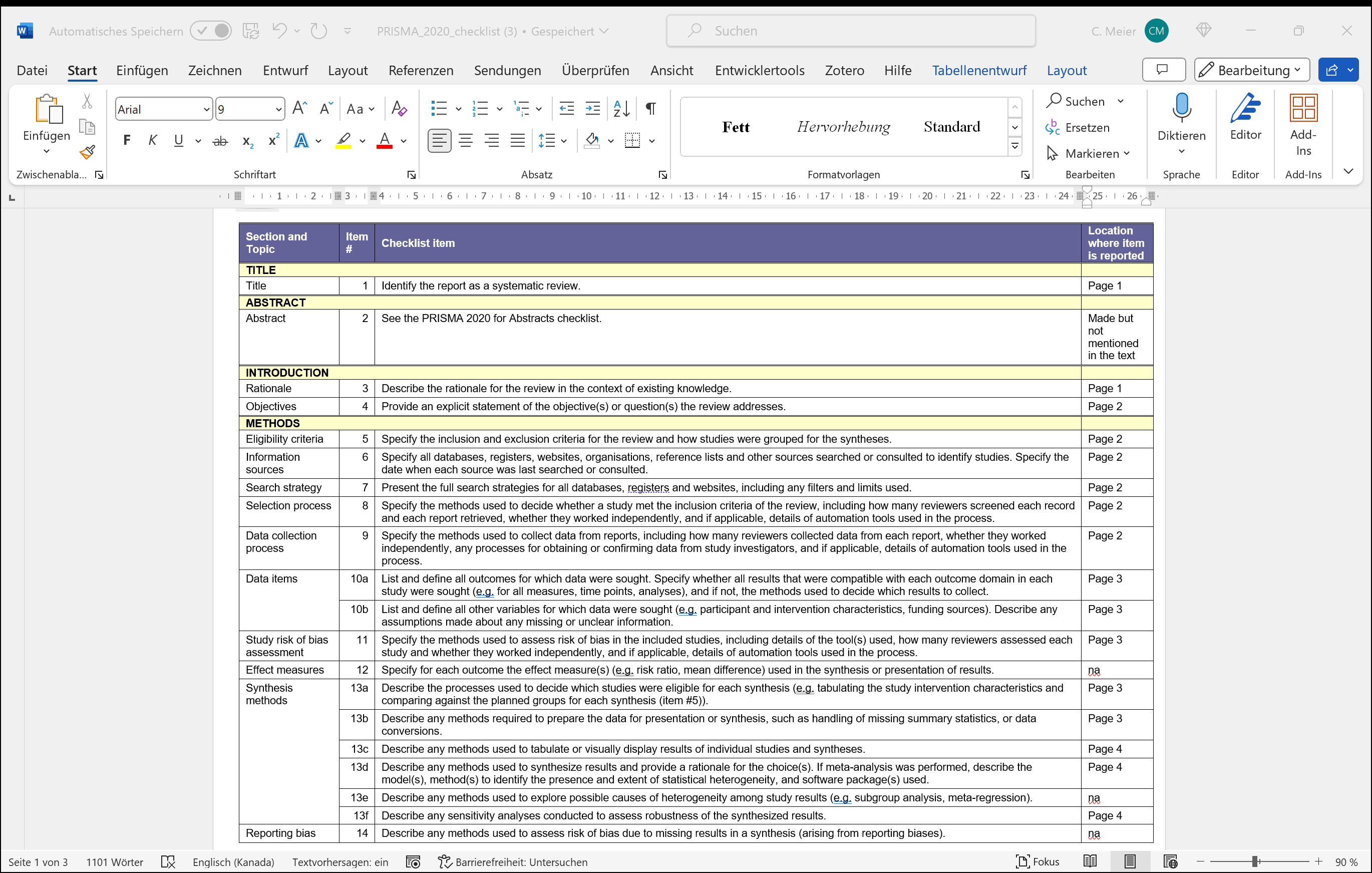


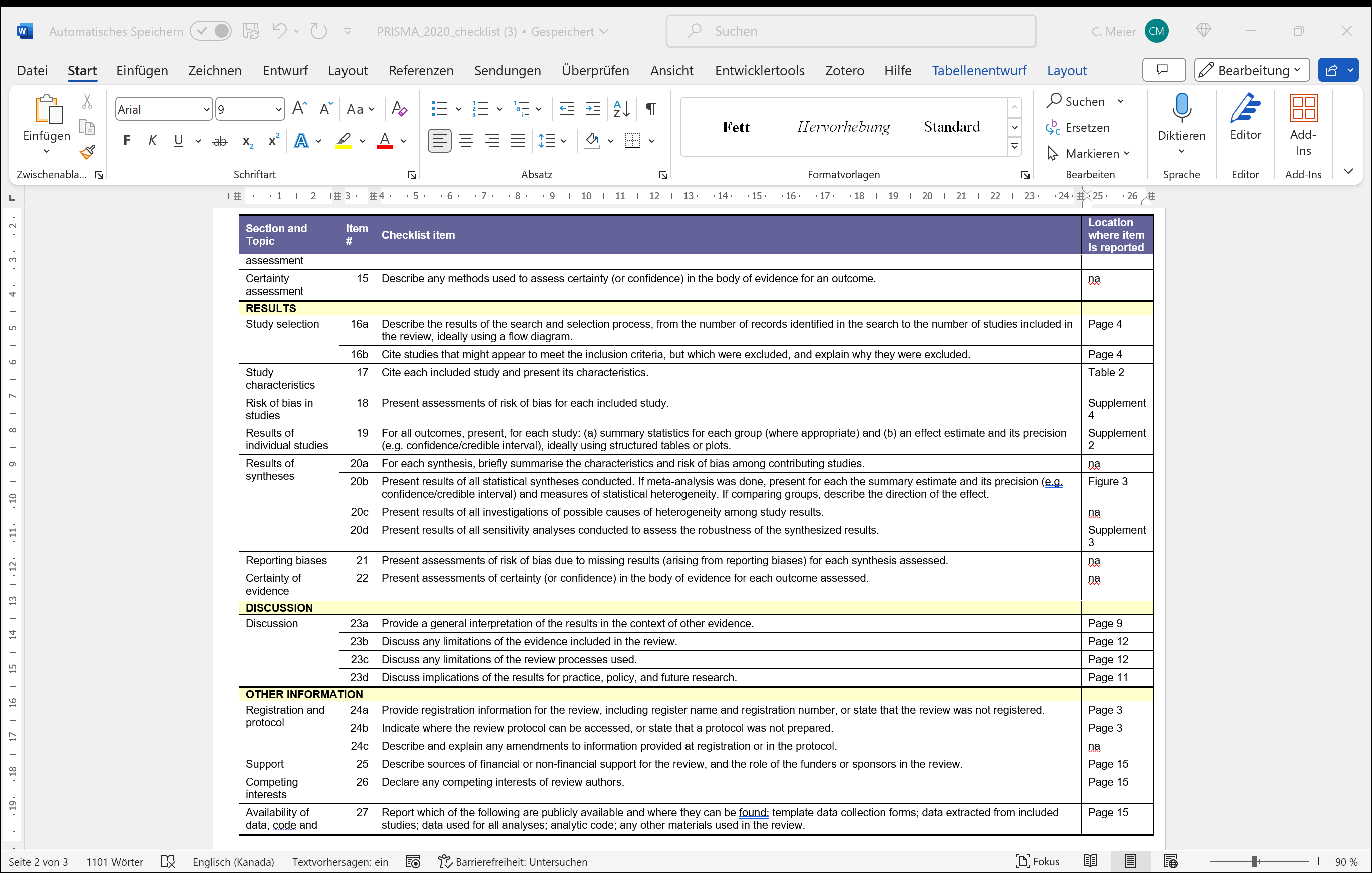


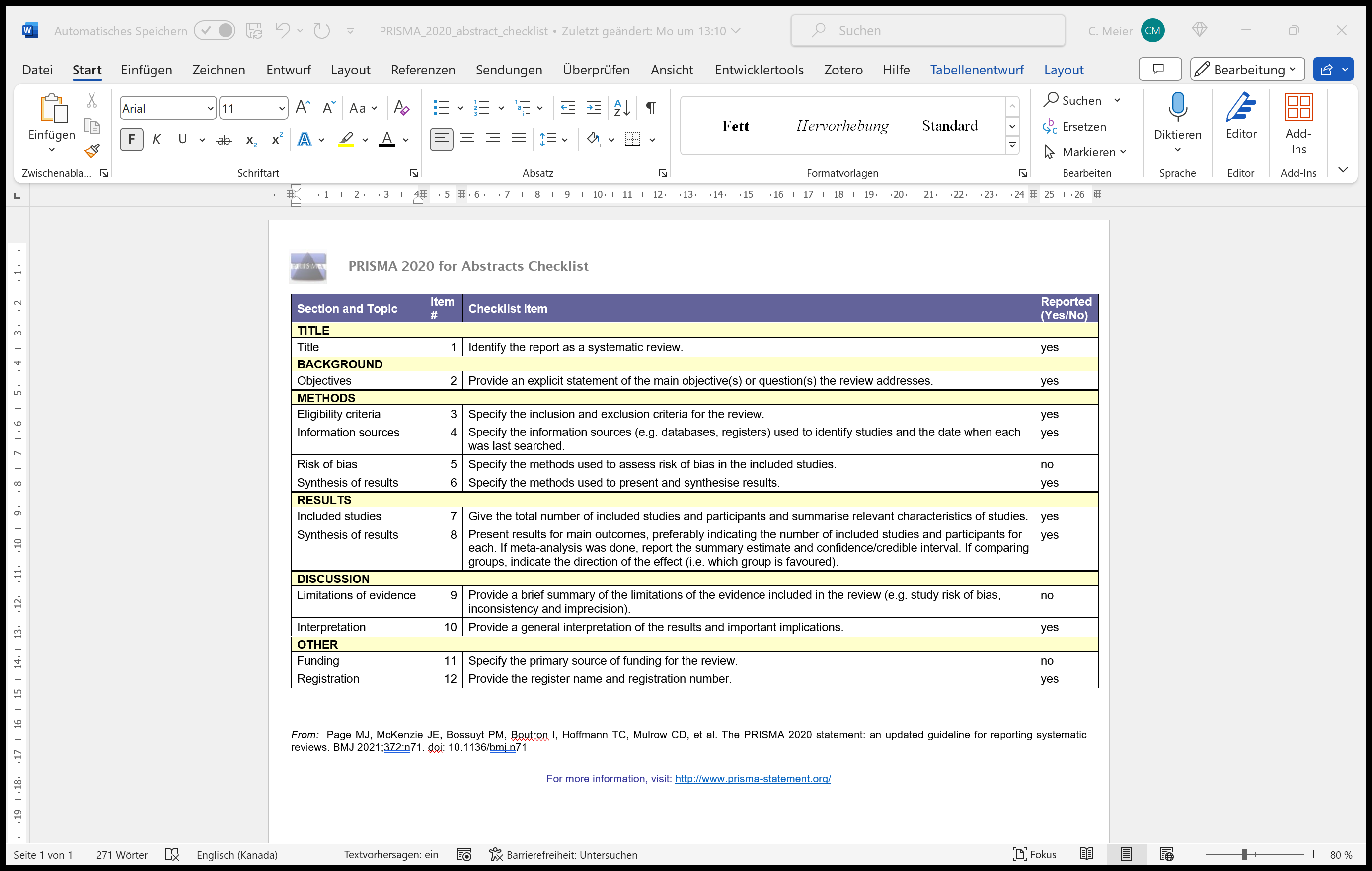

Supplement: euae165_Supplementary_Data [file euae165_supplementary_data.zip › Supplement 1 PRISMA check lists.docx]

Supplement 4: Conventional analysis of meta-data of the outcome "sensations"


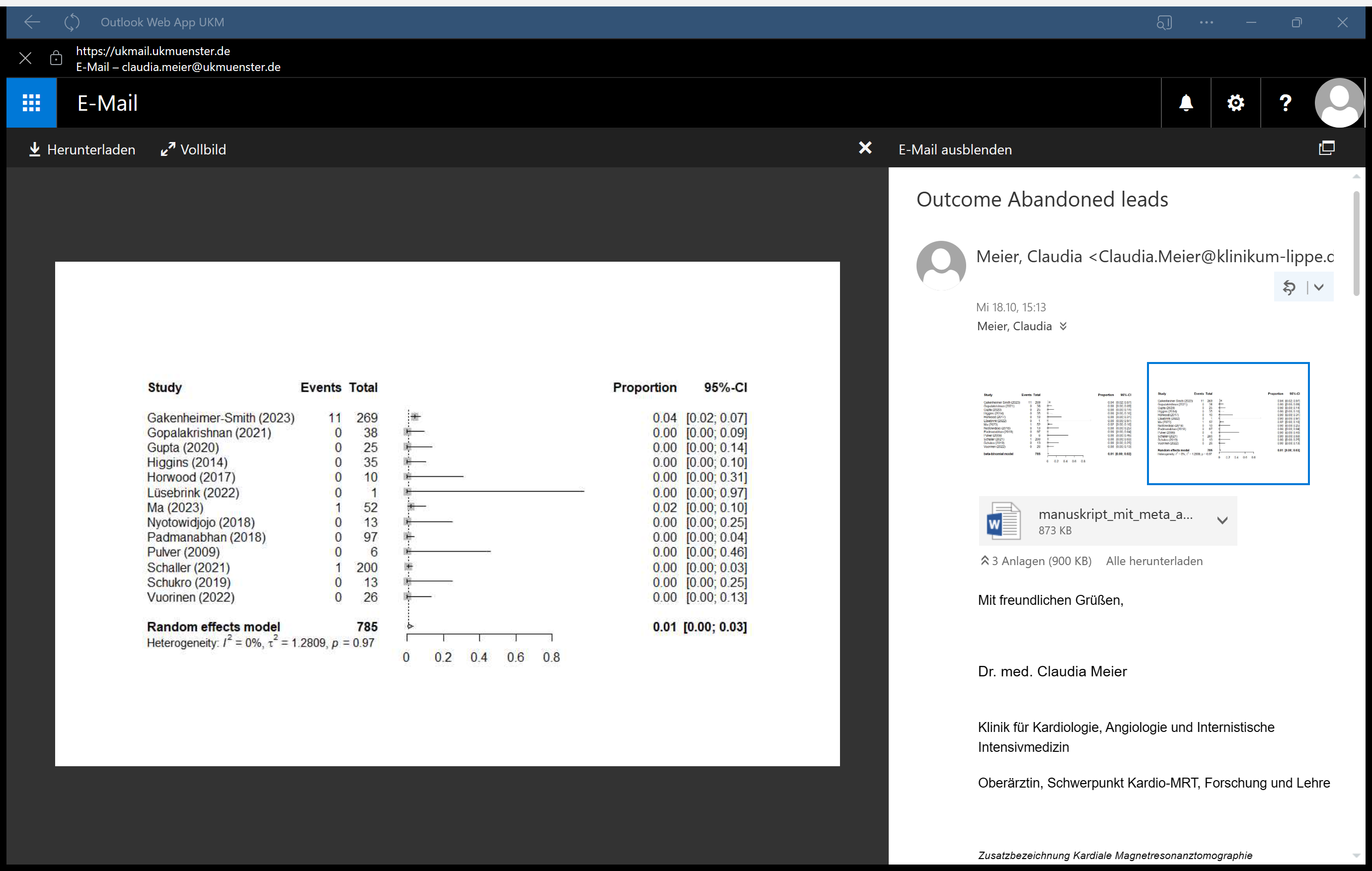

Supplement: euae165_Supplementary_Data [file euae165_supplementary_data.zip › Supplement 4 Forest plot conventional analysis.docx]

Supplement 5: Newcastle-Ottawa-scale


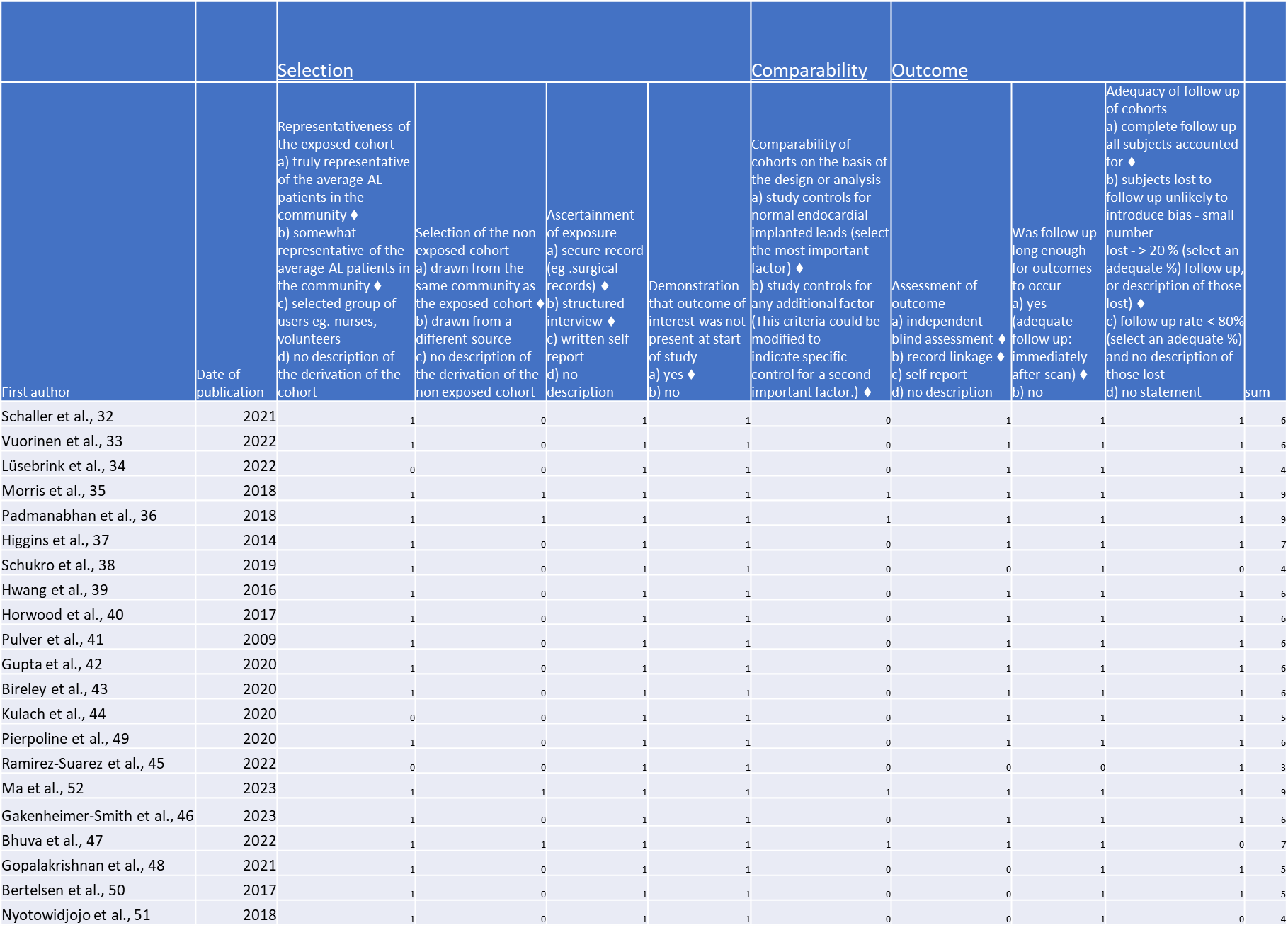

Supplement: euae165_Supplementary_Data [file euae165_supplementary_data.zip › Supplement 5 Newcastle-Ottawa-scale.docx]
